# Dual Targeting of MEK and PI3K Pathways Attenuates Established and Progressive Pulmonary Fibrosis

**DOI:** 10.1371/journal.pone.0086536

**Published:** 2014-01-27

**Authors:** Satish K. Madala, Ramakrishna Edukulla, Mukta Phatak, Stephanie Schmidt, Cynthia Davidson, Thomas H. Acciani, Thomas R. Korfhagen, Mario Medvedovic, Timothy D. LeCras, Kimberly Wagner, William D. Hardie

**Affiliations:** 1 Division of Pulmonary Medicine, Cincinnati Children’s Hospital Medical Center, Cincinnati, Ohio, United States of America; 2 Division of Pulmonary Biology, Cincinnati Children’s Hospital Medical Center, Cincinnati, Ohio, United States of America; 3 Laboratory for Statistical Genomics and Systems Biology, University of Cincinnati, Cincinnati, Ohio, United States of America; French National Centre for Scientific Research, France

## Abstract

Pulmonary fibrosis is often triggered by an epithelial injury resulting in the formation of fibrotic lesions in the lung, which progress to impair gas exchange and ultimately cause death. Recent clinical trials using drugs that target either inflammation or a specific molecule have failed, suggesting that multiple pathways and cellular processes need to be attenuated for effective reversal of established and progressive fibrosis. Although activation of MAPK and PI3K pathways have been detected in human fibrotic lung samples, the therapeutic benefits of in vivo modulation of the MAPK and PI3K pathways in combination are unknown. Overexpression of TGFα in the lung epithelium of transgenic mice results in the formation of fibrotic lesions similar to those found in human pulmonary fibrosis, and previous work from our group shows that inhibitors of either the MAPK or PI3K pathway can alter the progression of fibrosis. In this study, we sought to determine whether simultaneous inhibition of the MAPK and PI3K signaling pathways is a more effective therapeutic strategy for established and progressive pulmonary fibrosis. Our results showed that inhibiting both pathways had additive effects compared to inhibiting either pathway alone in reducing fibrotic burden, including reducing lung weight, pleural thickness, and total collagen in the lungs of TGFα mice. This study demonstrates that inhibiting MEK and PI3K in combination abolishes proliferative changes associated with fibrosis and myfibroblast accumulation and thus may serve as a therapeutic option in the treatment of human fibrotic lung disease where these pathways play a role.

## Introduction

Fibrotic lesions in the lung cause distortion of pulmonary architecture and interruption of gas exchange that ultimately can result in death [1]. Pulmonary fibrotic lesions are characterized by abnormal accumulation of mesenchymal cells accompanied by excessive deposition of extracellular matrix proteins such as collagen and fibronectin [1], [2]. Repetitive injury in the lung and genetic mutations are known to cause intrinsic changes in mesenchymal and epithelial cells, including cell migration, invasion, protection from apoptosis, and proliferation [3]–[8]. These processes are dependent on multiple signaling pathways that are dysregulated in injured epithelium and extracellular matrix (ECM)-producing mesenchymal cells [9], [10]. Therefore, successful therapies to either prevent the progression of fibrosis or reverse existing fibrotic disease likely need to target many of the signaling pathways that maintain fibrotic responses in the lung.

Fibroblast foci are clusters of fibroblasts and myofibroblasts that lie in continuity with established fibrosis, a characteristic histologic feature of pulmonary fibrosis especially idiopathic pulmonary fibrosis (IPF), and are thought to be central in mediating the progression of lung disease [11]. A three-dimensional reconstruction of the IPF lung demonstrates that fibroblast foci are at the leading edge of a complex polyclonal reticulum that extends from the pleura into the underlying parenchyma [12]. The concept of lung fibrosis as a neoproliferative process is further supported from studies in fibroblast cell lines from patients with IPF and other interstitial fibrotic diseases demonstrating that these cells possess intrinsic characteristics causing them to proliferate and survive better than normal fibroblasts [13], [14]. Thus, therapeutic strategies targeting cell proliferation may be effective in preventing fibroproliferative disorders in the lung.

Several growth factors and their downstream signaling pathways that maintain tumors are also activated in both human and mouse models of pulmonary fibrosis [15]–[18]. Receptor tyrosine kinases (RTK) are high-affinity cell-surface receptors for many polypeptide growth factors and cytokines, including ligands of the epidermal growth factor receptor (EGFR) and platelet-derived growth factor (PDGF), basic fibroblast growth factor, and vascular endothelial growth factor receptors [19], [20]. Following RTK activation, the cascade of subsequent signaling events activates multiple kinase pathways, including both the mitogen activated protein kinases (MAPK) and phosphatidylinositide 3-kinase (PI3K) pathways [9]. These two pathways have been shown to control cellular processes associated with fibrosis, including cell proliferation, growth, migration, and protection from apoptosis [21]–[23]. The relevance of MAPK and PI3K pathways in mediating fibrotic disease is further supported by studies of lung biopsies from patients with IPF, which demonstrate increased levels of signaling intermediates of the MAPK and PI3K pathways compared with normal lungs [16], [18], [24]. Furthermore, fibroblasts isolated from patients with IPF demonstrate altered negative regulation of PI3K leading to increased kinase activity and associated with significantly increased proliferation [24]. Collectively, emerging clinical data support a role for both MAPK and PI3K pathways in pulmonary fibrosis [2], [9], [25]. However, the crosstalk or functional synergisms that exist between MAPK and PI3K pathways in maintaining fibrotic lesions is not well understood.

We previously have generated doxycycline (Dox)-regulatable transgenic mice overexpressing the EGFR ligand, transforming growth factor-alpha (TGFα), under control of the lung epithelial-specific 2.3-kb rat Clara cell secretory protein (CCSP) gene promoter [26]. When CCSP/TGFα mice are administered Dox, the mice develop progressive pulmonary fibrosis characterized by specific phenotypes observed in human fibrotic disease, including epithelial and mesenchymal proliferation with myofibroblast differentiation, progressive migration of fibrotic lesions from the pleura into the interstitium, extracellular matrix deposition, severe restrictive changes in lung mechanics, and secondary pulmonary hypertension [21], [26]. Notably, signaling intermediates for the both MAPK and PI3K pathways are elevated during the progression of fibrosis in CCSP/TGFα mice, and we have previously demonstrated that specific pharmacologic inhibition of either the MEK or PI3K successfully prevented the initiation of fibrosis [15]. Furthermore, fibrotic progression was inhibited when the inhibitors were administered as a rescue therapy, although disease was not completely reversed [15].

The purpose of this study was to test the hypothesis that combined inhibition of MEK and PI3K signaling pathways would be more effective in treating established and progressing fibrosis in TGFα model than inhibiting either pathway alone. This study also examined transcriptional and fibroproliferative changes in the drug treated groups to determine overlapping or unique functions of each pathway in the maintenance of established and progressive fibrosis in the lungs.

## Materials and Methods

### Transgenic Mice and Administration of Inhibitors

All mice were derived from the FVB/NJ inbred strain. TGFα-transgenic mice were generated and maintained as described previously [17], [27]. Mice were housed under specific pathogen-free conditions, and protocols were approved by the Institutional Animal Use and Care Committee of the Cincinnati Children’s Hospital Research Foundation. To induce TGFα expression, Dox (Sigma, St. Louis, MO, USA) was administered in food (62.5 mg/kg). Stock solutions of AZD6244 (ARRY-142886 or ARRY) were prepared in 0.5% methocellulose/0.4% Tween 80 solution. The PI3K inhibitor, PX-866 (Oncothyreon Inc., Seattle, WA), was suspended in 5% ethanol to make a 5 mg/ml stock solution. Mice were anesthetized (isoflurane; Abbott Labs, Chicago, IL, USA), and sterile vehicle (solution used to prepare ARRY) or inhibitors were administered by gavage using a 20-gauge feeding catheter (Harvard Apparatus, Holliston, MA, USA). Dosing throughout the study was based on original baseline animal weights and not adjusted for weight changes. Mice were treated with ARRY once daily and PX-866 every other day for up to 4 weeks. Control mice were treated with similar volumes of vehicle, and our previous studies demonstrate that treatment with vehicle or Dox alone had no significant effect on TGFα-driven fibrosis [15], [17].

### Measurements of Lung Histology, Fibrosis Score, and Pleural Thickness

After removal, lungs were immediately inflation fixed using 10% neutral buffered formalin. Lung tissue sections were prepared and stained with Masson Trichrome as described previously [15]. The pleural thickness was measured by histomorphometric measurement on stained lung sections. Five random measures per lung section were obtained for each animal using a Leica DM2700 M bright field microscope (Leica Microsystems, Buffalo Grove, IL, USA). High-magnification images (40X) were captured with a 3CCD color video camera and analyzed using MetaMorph imaging software (v6.2; Molecular Devices, Sunnyvale, CA, USA). Pleural thickness was measured using the measured distance function of MetaMorph.

### Immunohistochemistry

For immunohistochemical detection of proliferation, paraffin-embedded lung sections were immunostained with anti-mouse Ki67 antigen (Dako, Glostrup, Denmark). The total numbers of Ki67-staining nuclei were counted, as well as the total number of nuclei in ten randomly selected uniform fields (26.2 mm^2^) that encompassed alveolar, pleural, and adventitial regions of the lung for each mouse. The proliferation index was determined by counting the total number of Ki67-staining nuclei and dividing by the total number of nuclei, in each field. The lung sections were immunostained for alpha smooth muscle actin as previously described [17].

### Hydroxyproline Assay

The total lung hydroxyproline levels were quantified using a method previously described [28]. In brief, the left lobe of lung was weighed and hydrolyzed in 4 ml of 6 N HCl overnight at 110°C. Hydrolyzed samples were neutralized with 1 N NAOH. For colorization, chloramine-T (0.05 M) and the aldehyde-perchloric acid reagent were added, and samples were placed in a hot-water bath at 60°C for 25 min. Standard hydroxyl-L-proline (EMD) solutions were prepared and applied identically to the samples to read at 550 nm for samples and standards. Sample concentrations were determined from the standard curve.

### Pulmonary Mechanics

Lung mechanics were assessed on mice using a computerized Flexi Vent system (SCIREQ, Montreal, Canada) as described previously [15].

### RNA Preparation and Real-Time PCR

Total RNA was extracted from lung tissue and cells using the RNeasy Mini Kit from Qiagen (Qiagen Sciences, Valencia, CA, USA) as described previously [15]. Real-time primer sequences used were HPRT 5′-GCCCTTGACTATAATGAGTACTTCAGG-3′ (forward),5′-TCAACTTGCGCTCATCTTAGG-3′ (reverse); MYCN 5′-AGCACCTCCGGAGAGGATAC-3′ (forward), 5′-CCACATCGATTTCCTCCTCT-3′ (reverse); CDK4 5′-AGAGCTCTTAGCCGAGCGTA-3′ (forward), 5′-TTCAGCCACGGGTTCATATC-3′ (reverse).

### Whole-transcriptome Shotgun Sequencing (RNA-Seq) and Heat Maps

From each experimental group, four lung tissue samples were sequenced using an Illumina HiSeq-1000 Sequencer (Illumina Inc., San Diego, CA). An in-house analytical pipeline was created to align reads to the reference genome, perform quality control, and conduct statistical analyses to identify and cluster differentially expressed genes. Using TopHat aligner [29], reads were aligned to the reference genome based on the current gene definitions and those aligned with low confidence were filtered out using SAM tools [30], which resulted in approximately 25 million reads on average per sample. The number of reads (read counts) aligning to the gene’s coding region was summarized using ShortRead [31] and various R- Bioconductor [32] packages (IRanges, GenomicRanges, Biostrings, Rsamtools).

To identify differentially expressed genes between Day 0 (untreated) and Week 8 (treated on Dox) samples, statistical analysis was performed using the DESeq Bioconductor package [33] that uses a statistical model based on negative-binomial distribution of the read counts. Statistically significant genes were selected based on a *P*-value cut-off of 0.05 as well as a two-fold change (up or down), resulting in 1790 genes in total. Using this set of significant genes, hierarchical clustering on the log-transformed read counts normalized for different lengths of gene-coding regions (RPKM values) [34] was performed for ARRY-, PX-866-, and ARRY/PX-866-treated groups, and a heat map was generated. Genes involved in the proliferation and survival of lung cells were identified using a cellular functional analysis tool available in Ingenuity Pathway Analysis (Ingenuity Systems, Redwood City, CA, USA). Complete RNA-Seq data is available at gene expression omnibus (GEO) database (http://www.ncbi.nlm.nih.gov/geo/query/acc.cgi?acc=GSE52854; accession number: GSE52854).

### Western-Blot Analysis

Lung homogenates were processed for Western blot and quantified using the PhosphorImager software volume-integration function, Imagequant 5.2 (Molecular Dynamics, CA, USA) as described previously [15]. Primary antibodies used included those for GAPDH (Bethyl Labs, Montgomery, TX, USA), total Erk1/2 and phosphorylated Erk1/2 (Thr 202/Tyr 204), and total S6 and phosphorylated S6 (Ser 235/236).

### 3D Collagen Gel Contraction Assay

Collagen gel contraction was performed as described elsewhere with few modifications [35]. Rat tail type 1 collagen dissolved in 0.2% acetic acid (6 mg/ml) for 5–7 days at 4°C and diluted with equal volume of sterile water to make 3 mg/ml of collagen and 0.1% acetic acid. Media containing primary fibroblasts (5×10^4^ cells/well) and collagen (3 mg/ml) were combined at a ratio of 2∶1 to obtain collagen gels with cells, and plated in 24 well tissue culture plates. The next day gels were gently taken out of 24 well plates and placed them in 6 well plates containing media, ARRY (0.1 µM), PX866 (0.1 µM) or both the inhibitors in combination. Culture media was changed on every day and also gel images taken to quantify the surface area of collagen gels.

### Statistics

All data were analyzed using Prism (Version 5; GraphPad, La Jolla, CA, USA). One-way ANOVA with Tukey’s Multiple Comparison post-test was used to compare different experimental groups, and data were considered statistically significant for *P* values less than 0.05.

## Results

### Combined Therapy Using Both MEK and PI3K Inhibitors is More Effective in Reversing Collagen Accumulation in the Lungs than Using Either Inhibitor Alone

**Figure 1:**
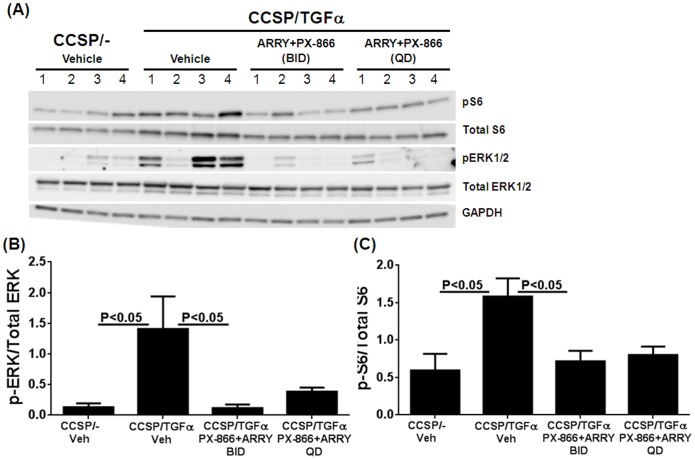
Effect of in in vivo inhibition of MEK and PI3K in combination on ERK and S6 phosphorylations. (A) CCSP/− and CCSP/TGFa mice on Dox for 2 weeks were co-treated with PX-866 (3 mg/kg, every other day) and ARRY (37.5 mg/kg) either once a day (QD) or twice a day (BID) for 7 days. Total lung lysates that represent four separate mice in each group were analyzed by immunobloting. Western blot analysis was used to determine levels of phosphorylated ERK (B) and phosphorylated S6 (C) that normalized to the total ERK and S6 protein in lung homogenates, respectively. Data are means ± SEM, and statistical significance between groups was measured using one-way ANNOVA (n = 4/group).

To evaluate the effects of combined therapy with ARRY and PX-866, we used TGFα transgenic mice which develop progressive and severe pulmonary fibrosis [26]. Our previous studies determined that PX-866 [3 mg/kg, every other day] and ARRY [37.5 mg/kg, twice a day (BID)] inhibited fibrosis when administered alone compared to vehicle-treated mice [15], [17]. However, the effective doses of these inhibitors required to inhibit both MEK and PI3K signaling pathways when the inhibitors are combined were unknown. To establish the doses of combination therapy effective to inhibit both pathways, TGFα transgenic mice were treated with Dox for 2 weeks to induce TGFα expression and randomized into three groups receiving either vehicle or PX-866 (3 mg/kg, every other day) combined with ARRY (37.5 mg/kg), either once a day (QD) or twice a day (BID) for 7 days. The activation of downstream signaling intermediates for the PI3K and MAPK pathways were analyzed in total lung lysates. Combined therapy of PX-866 with ARRY administered either once a day or twice a day was equally effective in suppressing the activation of both pERK and pS6 compared to vehicle-treated mice (Figure 1). As once daily dosing of ARRY was sufficient to attenuate the activation of ERK we administered this dose in our subsequent long-term fibrosis-reversal studies to reduce the potential for toxicity when using dual inhibitors.

**Figure 2:**
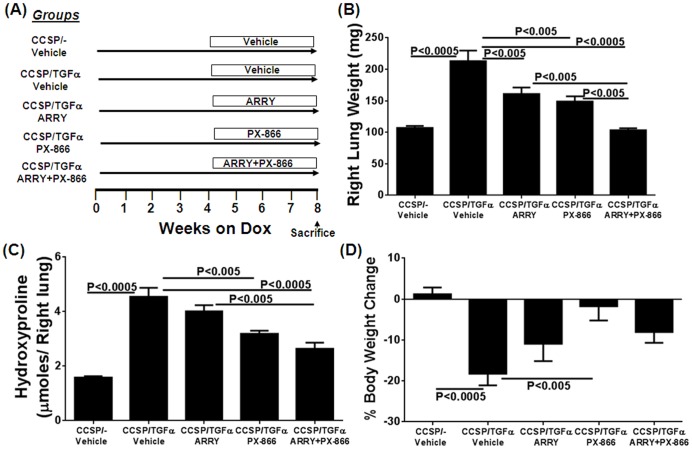
Inhibition of MEK and PI3K alone or in combination alters lung weights and collagen accumulation during TGFα-induced pulmonary fibrosis. (A) The treatment protocol is represented schematically. Controls included CCSP/− and CCSP/TGFα mice treated with vehicle for the last 4 weeks while remaining on Dox a total of 8 weeks. (B) Changes in the right lung weights of mice treated with vehicle or inhibitors. (C) Changes in the right-lung hydroxyproline levels of mice treated with vehicle or inhibitors. Data are means ± SEM, and statistical significance between groups was measured using one-way ANNOVA (n = 8–12/group). (D) Percent change in body weights of mice treated with vehicle or inhibitors. Data are means ± SEM, and statistical significance between groups was measured using one-way ANNOVA (n = 8–12/group).

To determine whether combined therapy with ARRY and PX-866 influences the progression of fibrosis when fibrosis is already established (following 4 weeks of Dox), TGFα transgenic mice were treated with ARRY and PX-866, either alone or in combination, while remaining on Dox for an additional 4 weeks (Figure 2A). Total lung weights and hydroxyproline levels were increased more than two-fold in TGFα transgenic mice treated with vehicle and Dox for 8 weeks compared with CCSP/− control mice on Dox (Figure 2B and 2C). Total lung weights were reduced in TGFα transgenic mice treated with either ARRY or PX-866 alone compared with vehicle-treated TGFα transgenic mice (Figure 2B). Combination therapy further reduced total lung weights compared with TGFα transgenic mice treated with either inhibitor alone (Figure 2B). Similarly, hydroxyproline levels were significantly reduced in mice treated with both PX-866 or combination therapy compared to TGFα transgenic mice treated with vehicle and combination therapy reduced hydroxyproline levels compared to ARRY-treated mice (Figure 2C). CCSP/TGFα mice treated with 8 weeks of Dox and administered vehicle show a significant loss in body weights compared to vehicle treated CCSP/− mice. Whereas, CCSP/TGFα mice treated with PX-866 alone showed a significant protection in body weight loss, while CCSP/TGFα mice treated with ARRY or combination therapy show reduced but not significant protection in body weight loss (Figure 2D).

### Combined Therapy Using Both MEK and PI3K Inhibitors is More Effective in Reversing Fibrotic Changes in the Lungs than Using Either Inhibitor Alone

**Figure 3:**
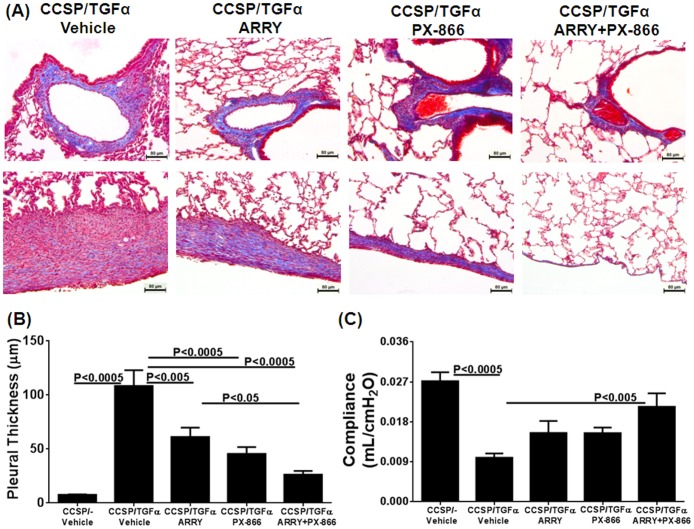
Inhibition of MEK and PI3K alone or in combination alters collagen accumulation and pleural thickening during TGFα-induced pulmonary fibrosis. (A) Representative photomicrographs of lung tissues stained with Masson Trichrome for each treatment group. Upper panel: area of airways, vessels, and parenchyma. Lower panel: pleural regions. Scale bar, 80 µm. (B) Pleural thickness of lung tissues stained with Masson Trichrome for treatment groups. (D) Inhibition of MEK and PI3K alone or in combination alters lung compliance during TGFα-induced pulmonary fibrosis. Data are means ± SEM, and statistical significance between groups was measured using one-way ANNOVA (n = 8–12/group).

Lung sections were stained with Masson Trichrome to visualize fibrotic changes in the lungs of CCSP/TGFα mice treated with either vehicle or inhibitors (Figure 3A). Overexpression of TGFα resulted in collagen deposition and thickening of adventitia, interstitium, and pleura in CCSP/TGFα mice treated with vehicle and Dox for 8 weeks (Figure 3A). Combination therapy resulted in notable reductions in collagen deposition in the fibrotic lesions, especially in the pleura, compared to CCSP/TGFα mice treated with either individual inhibitors or vehicle (Figure 3A). To quantify fibrotic changes in the pleura of lung sections, we measured the thickness of the pleura of vehicle- and inhibitor-treated groups (Figure 3B). CCSP/TGFα mice treated with either ARRY or PX-866 alone demonstrated reduced pleural thickness compared with vehicle-treated CCSP/TGFα mice (Figure 3B). Combined therapy further reduced the pleural thickness and was significantly reduced compared with ARRY-treated mice (Figure 3B). CCSP/TGFα mice treated with vehicle and Dox for 8 weeks show significant changes in the lung compliance compared with CCSP/− control mice (Figure 3C). Lung compliance was only improved in mice receiving combination therapy (Figure 3C). No deaths occurred in the controls, with one death in the vehicle or inhibitors-treated CCSP/TGFα mice on Dox. These deaths were unrelated to the fibrosis as they occurred early in the study before significant fibrosis develops. In addition treatment of mice with inhibitors alone or in combination did not lead to any changes in serum alanine aminotransferase (ALT) or creatinine (Cr) (Figure S1).

**Figure 4:**
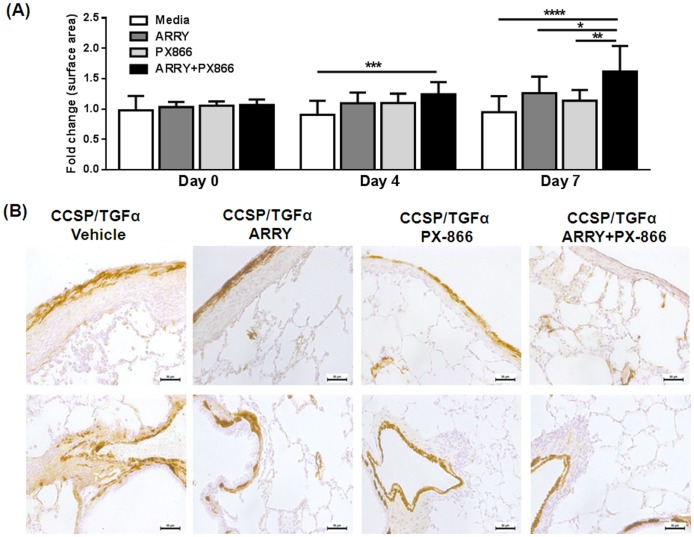
Inhibition of MEK and PI3K in combination alters contraction of collagen gels and SMA deposition in the lungs. (A) Primary fibroblasts from the lung cultures of CCSP/TGFα transgenic mice on Dox for 4 wks were platted in 3D collagen gels for 4 or 7 days with media, ARRY, PX866 or inhibitors combined. Images were taken to quantify the changes in the surface of 3D collagen gels (B) Representative photomicrographs of lung tissues for each treatment group (n = 4/group) demonstrate changes in SMA staining in the fibrotic lesions of the pleura (upper panel) and adventitia (lower panel).

To examine mechanisms whereby inhibitors alone or in combination prevent the progression of fibrotic disease in the CCSP/TGFα model, a gel contraction assay was performed on primary fibroblasts from the lungs of CCSP/TGFα mice on Dox for 4 weeks. Gels receiving inhibitors alone or in combination demonstrated significantly less contraction than untreated gels with the greatest improvement seen in fibroblasts receiving combination treatment (Figure 4A). One possible mechanism for improved contraction is reduced transformation of myofibroblasts. To determine changes in myofibroblasts in vivo, changes in the intensity of contractile protein smooth muscle α-actin (SMA) deposition were assessed in the pleural and advential areas of lung sections. Less SMA staining was seen in mice receiving inhibitors alone or in combination than vehicle-treated mice with the least staining seen in mice receiving combination treatment (Figure 4B).

### Combined Therapy using both MEK and PI3K Inhibitors is more Effective to Attenuate Cell Proliferation than using Either Inhibitor Alone

**Figure 5:**
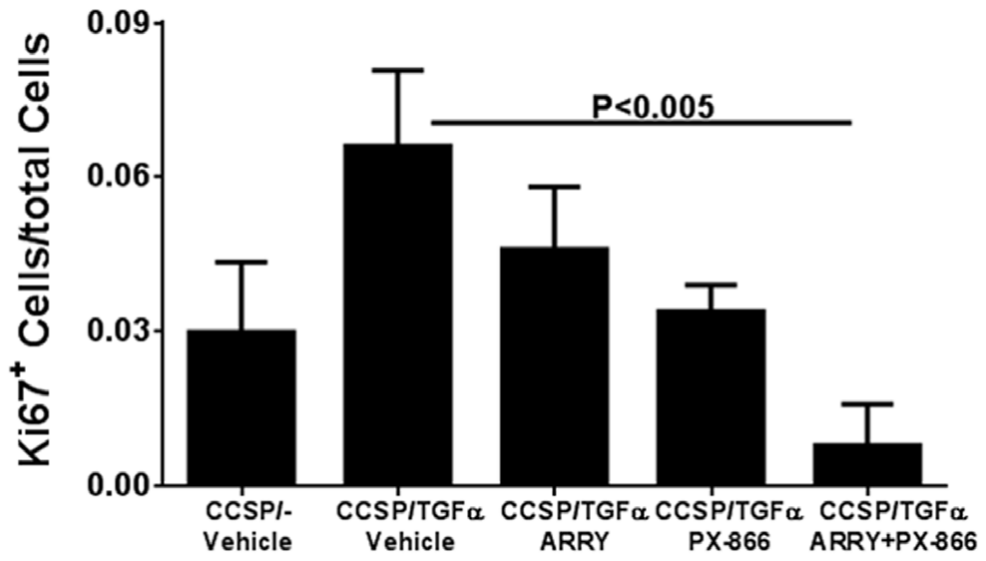
Inhibition of MEK and PI3K alone or in combination alters proliferating cells in the lung during TGFα-induced pulmonary fibrosis. Changes in the number Ki67 positive cells of mice in each group on Dox for 8± SEM and statistical significance between groups was measured using one-way ANNOVA (n = 4–5/group).

The excessive proliferation of mesenchymal and epithelial cells has been shown to contribute to progressive fibrosis in both human and mouse lungs. To determine the role of MEK and PI3K inhibitors in proliferation, we quantified changes in proliferation by counting Ki67-positive cells in lung sections. Overexpression of TGFα mice for 8 weeks had doubled cells staining for Ki67 compared with vehicle-treated CCSP/− mice. TGFα transgenic mice treated with either ARRY or PX-866 alone had reduced Ki67 staining compared with vehicle-treated TGFα transgenic mice (Figure 5 and Figure S2). Combined treatment resulted in a synergistic effect by further reducing the number of proliferating cells compared with vehicle-treated TGFα transgenic mice on Dox (Figure 5).

### Combined Therapy Using Both MEK and PI3K Inhibitors is More Effective in Reversing the Expression of Genes Associated with Proliferation and Fibrosis than Using Either Inhibitor Alone

**Figure 6:**
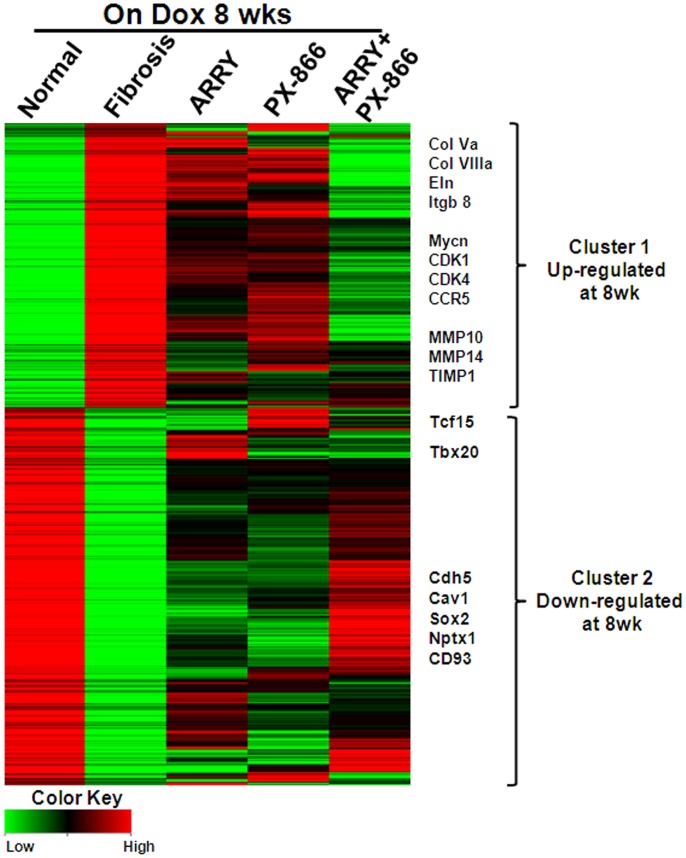
Inhibition of MEK and PI3K alone or in combination alters the transcriptome of the lung during TGFα-induced pulmonary fibrosis. Heatmap depicting expression levels of total lung genes with at least a two-fold change from CCSP/− control mice on Dox for 8 wks. Differentially expressed genes (up- or down-regulated) by ≥ two-fold in the fibrosis, ARRY, PX-866, ARRY/PX-866 in combination treatment groups are shown. Expression levels of these genes were either maintained or enriched in the combination group as compared to both single inhibitors. Several known fibrotic genes that are significantly down regulated in combined therapy are highlighted using their gene symbols.

To identify unique and overlapping molecular networks by which MEK and PI3K pathways regulate proliferation of cells and fibrosis in the lung, we assessed gene-expression changes in the lungs of TGFα mice treated with vehicle or inhibitors using next-generation sequencing. As shown in Figure 6, there were two clusters of differentially-expressed genes in the fibrotic lungs of TGFα mice on Dox for 8 weeks compared to control mice. The list of genes in Cluster 1 and Cluster 2 represent genes that are upregulated (769 genes) or down regulated (1021 genes) approximately two fold or more following TGFα overexpression for 8 weeks. Treatment with either ARRY or PX-866 alone had a significant effect in altering expression levels for gene transcripts that are up regulated or down regulated in the fibrosis group (Figure 6). Notably, combined ARRY/PX-866 therapy (Column 5, ARRY+PX-866) was more effective in reversing transcript expression levels for the majority of genes that were differentially expressed in the fibrosis group (Column 2, fibrosis). Genes with the largest decreases in Cluster 1 include extracellular matrix genes (collagens, elastin, and integrin genes), chemokines (CCL2 and CCR5), regulators of fibroproliferation (MYCN, FN1, AREG, and EREG), and proteases involved in ECM remodeling (MMP10, MMP14, and TIMP1) (Figure 6). Similarly, the expression of down regulated genes in the fibrosis group was reversed and was more similar to control mice than mice treated with dual inhibitors, and includes transcription factors and cadherins (Cdh5, Cav1, Sox2, and Nptx1) (Figure 6).

**Figure 7:**
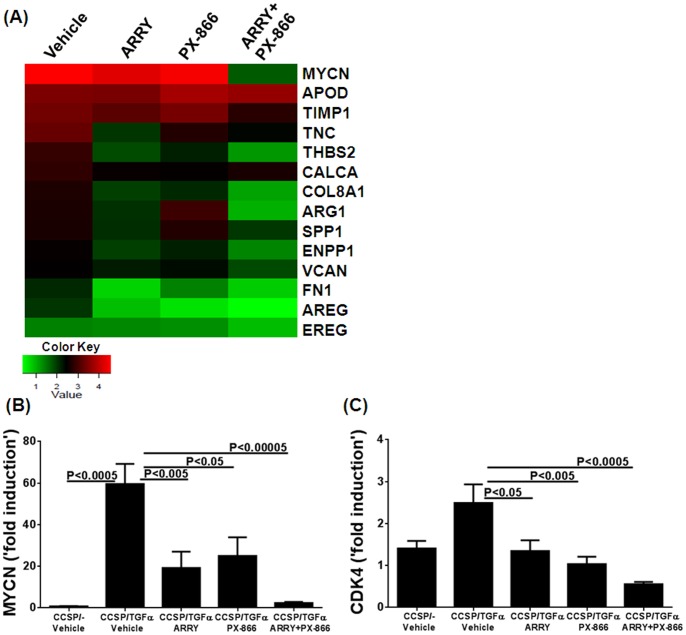
Inhibition of MEK and PI3K alone or in combination alters transcripts involved in the proliferation of cells in the lung during TGFα-induced pulmonary fibrosis. (A) Heatmap depicting expression levels of several top genes involved in proliferation, based on ingenuity pathway analysis. Up-regulated genes in the lungs of mice treated with vehicle, ARRY, PX-866, or ARRY/PX-866 in combination are shown. (B) MYCN transcript levels in the lungs of each treatment group on Dox for 8 wks, during which mice were treated with either vehicle or inhibitors for the last 4 wks (C) CDK4 transcript levels in the lungs of each treatment group on Dox for 8 wks, during which mice were treated with either vehicle or inhibitors for the last 4 wks. Data are means ± SEM, and statistical significance between groups was measured using one-way ANNOVA (n = 4/group).

The network of genes that contribute to the excessive proliferation of cells in fibrotic lesions is of major interest, since combined therapy was more effective in inhibiting proliferation of cells in the lung. Also, the gene targets regulated by both MEK and PI3K pathway-driven proliferation are largely unknown. Heat mapping was used to show the top genes that are differentially expressed and involved in cell proliferation, using ingenuity pathway analysis. As shown in Figure 7A, both the MEK and PI3K pathways altered the expression of the majority of genes involved in cell proliferation and fibrotic disease. One of the top genes in this list is the transcriptional regulator of proliferation, MYCN, which is highly induced in the lungs of fibrotic mice (Figure 7A and 7B). Notably, combined ARRY/PX-866 therapy was more effective in reducing transcripts for MYCN in the lungs compared to vehicle or single-inhibitor treatment (Figure 7A and 7B). The expression of cyclin-dependent kinases are critical for cell-cycle progression, and the combined therapy was most effective in reducing CDK4 transcript levels compared to vehicle treatment (Figure 7C).

## Discussion

Pulmonary fibrosis complicates a wide range of adult and pediatric diseases including autoimmune, inflammatory, and idiopathic disorders [2], [36]. In most of these progressive disorders the specific genetic or molecular etiology driving the pathologic fibrosis is unknown. However, lung fibrosis diseases share common pathologic dysregulated cellular processes such as cell growth and especially proliferation of mesenchymal cells [4], [5]. Thus, although the upstream genetic or molecular causes may not be known or amenable to therapy, focusing on the downstream cellular processes that ultimately lead to mesenchymal-cell accumulations may prove to be a novel and feasible therapeutic approach. The TGFα mouse model allows in vivo assessment of signaling pathways mediating pulmonary fibrosis. In this report, we show that combined inhibition of MEK and PI3K may represent a more effective preclinical therapy for established and progressive fibrosis than inhibition of either pathway alone.

Cellular pathways are highly integrated webs with multiple redundancies that may be activated in response to the inhibition of a signaling pathway [37], [38]. For this reason, combination therapies are often needed to effectively treat many tumors and infectious diseases. Similar findings from our study are illustrated by the direct measure of cellular proliferation of lung sections using Ki67 staining, whereby individual treatment using either MAPK or PI3K inhibitors attenuated the proliferation index. However, proliferation was further reduced when the inhibitors were combined, which suggests synergistic interactions between MAPK and PI3K pathways in altering lung-cell proliferation in a fibrotic disease. Our previous studies demonstrated that inhibition of MEK had no effect on the activation of Akt and its downstream target S6 [15]. Similarly, PX-866 administered alone had no effect on levels of pERK in the lungs of TGFα mice. This suggests that the limited effect of either inhibitor alone is due to the maintenance of fibrosis by other alternative pathways. In this study, the administration of dual inhibitors was effective in inhibiting the phosphorylation of both ERK and S6. Using RNA sequencing analysis, we identified several genes known to regulate cellular proliferation and growth downstream of the MAPK and PI3K pathways. One was the oncogenic transcription factor MYCN, which is a member of the MYC proto-oncogene family that also includes c-MYC and MYCL. Like other MYC proteins, MYCN encodes nuclear phosphoproteins that function as sequence-specific transcription factors and target a large number of genes, including many involved in the control of cell proliferation, growth and apoptosis [39]. Data from our study indicates that inhibition of both MEK and PI3K is required to suppress increases in MYCN transcripts induced in the fibrotic lungs of TGFα transgenic mice. Notably, average MYCN mRNA levels also are elevated in individuals with interstitial lung disease compared to controls and COPD (Figure S3). Our results extend these observations by showing that MYCN represents an important downstream target of both MEK and PI3K pathways in the lung. Our findings also demonstrate that the MEK and PI3K pathways converge to activate a common set of downstream genes that are highly integrated and therefore less likely to be responsive to individual pathway inhibition once disease is established.

Although fibrosis was reduced using combined treatment compared to single-inhibitor therapy, fibrosis endpoints in the combination-treatment group remained altered compared with non-fibrotic controls. We have previously demonstrated that hydroxyproline levels and pleural thickening remain elevated up to 18 weeks following extinction of TGFα overexpression [21]. Thus, a certain residual fibrosis would be expected following 4 weeks of treatment, and a longer treatment time would be necessary to determine whether combined treatment is sufficient to completely reverse fibrosis. Another alternative explanation is that other pro-fibrotic pathways are not inhibited by the use of combined MEK and PI3K inhibitors and these may include JAK/STAT and TGFβ/SMAD pathways. Both initiation and reversal of fibrosis involves the recruitment of inflammatory cells such as neutrophils, lymphocytes and macrophages [7]. In particular, our recent study suggests that bone marrow derived fibrocytes trigger fibroblast proliferation and accumulation in the fibrotic lesions [40]. Therefore, future studies will be needed to determine if MEK and PI3K inhibitors can alter the recruitment of inflammatory cells that participate in the initiation and resolution of fibrosis.

Several cancers are associated with mutations in the MAPK and/or PI3K pathways, and there are multiple pharmacologic agents that specifically target these pathways currently in advanced clinical trials. Since both MAPK and PI3K signaling pathways are elevated in fibrotic lung diseases, the findings of this study are potentially translational. Currently there are a number of clinical trials in oncology where both inhibitors are administered in combination, so safety data will be emerging on the feasibility of this approach for treating lung fibrosis [41]. However, targeted therapies may improve efficacy with less toxicity, but the effects are not durable [37], [38]. The dual inhibition of both MEK and PI3K pathways has been shown to exhibit favorable efficacy and be more effective in the long-term compared to single-pathway inhibitors, but may also cause greater toxicity in clinical settings [41]. In this study, dual targeting of MEK and PI3K showed no significant toxic effects, possibly due to use of lower doses of inhibitors. In summary, data from our study establishes that combined pharmacologic inhibition of the MAPK and PI3K pathways in the TGFα model provides more effective resolution of pulmonary fibrosis.and could potentially enhance the survival of patients with interstitial lung disease.

## Supporting Information

Figure S1 **Combined inhibition of MEK and PI3K pathway inhibitors had no severe toxic effects on kidney (creatinine) and liver (serum ALT) functions during TGFα-induced fibrosis.**
(PDF)

Figure S2 **Representative photomicrograph of lung tissue sections immunostained for Ki67 for each treatment group.** Scale bar, 100 µm.
(PDF)

Figure S3 **Expression of MYCN in the lungs of individuals with interstitial lung disease.** Average Mycn mRNA levels are elevated in individuals with interstitial lung disease compared to controls and COPD. Mycn expression data was extracted from mRNA expression catalogue available in the database of Lung Genomic Research Consortium (LGRC) (https://www.lung-genomics.org/research).
(PDF)

Data S1 (PDF)
