# Supplementary material for: Dual Targeting of MEK and PI3K Pathways Attenuates Established and Progressive Pulmonary Fibrosis
Source: PLoS One. 2014 Jan 27;9(1):e86536. doi: 10.1371/journal.pone.0086536 (PMC3903543; doi:10.1371/journal.pone.0086536)

Figure S1: Combined inhibition of MEK and PI3K pathway inhibitors had no severe toxic effects on kidney (creatinine) and liver (serum ALT) functions during TGFα-induced fibrosis.

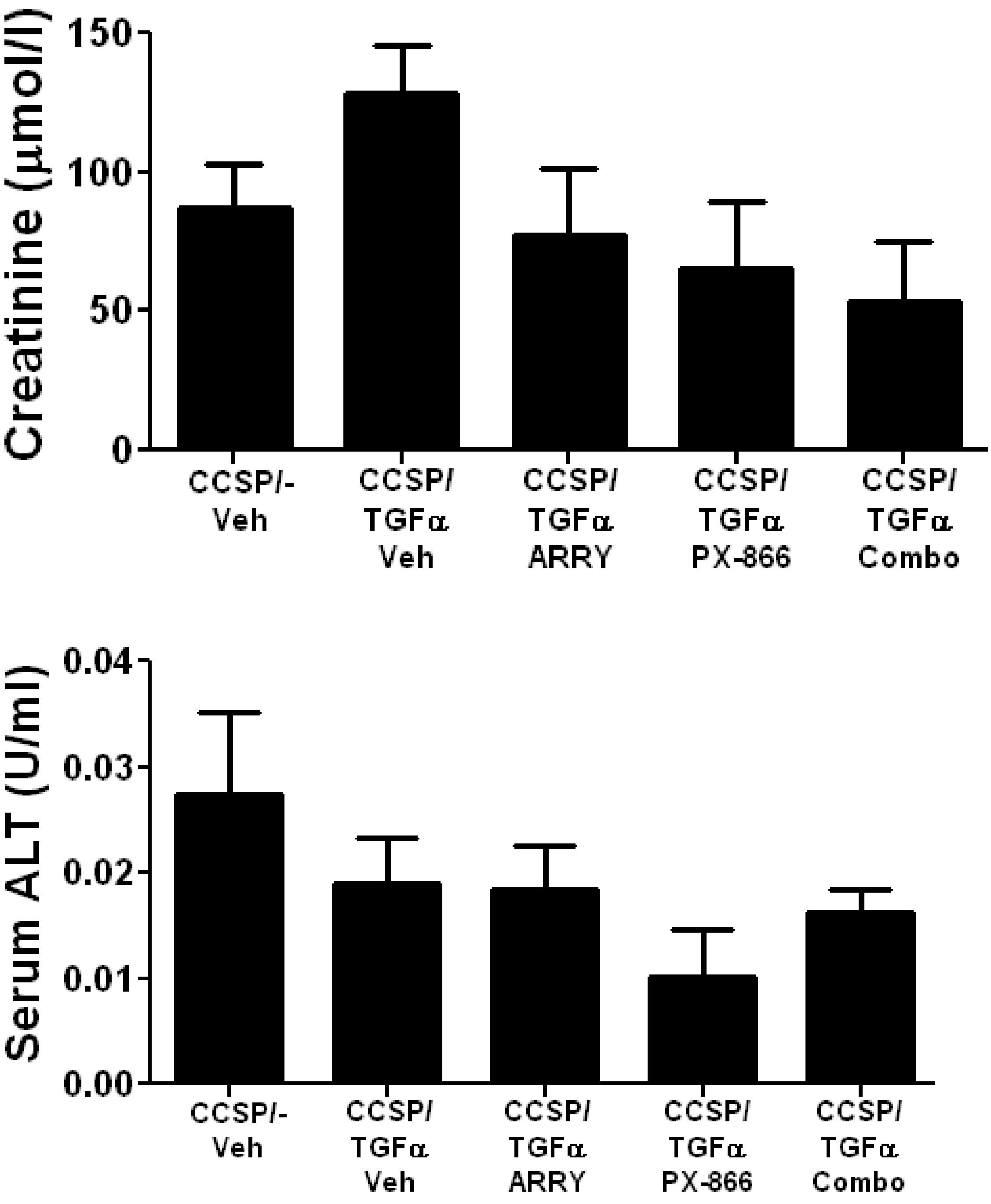

Figure S2: Representative photomicrograph of lung tissue sections immunostained for Ki67 for each treatment group. Scale bar, 100 μm.

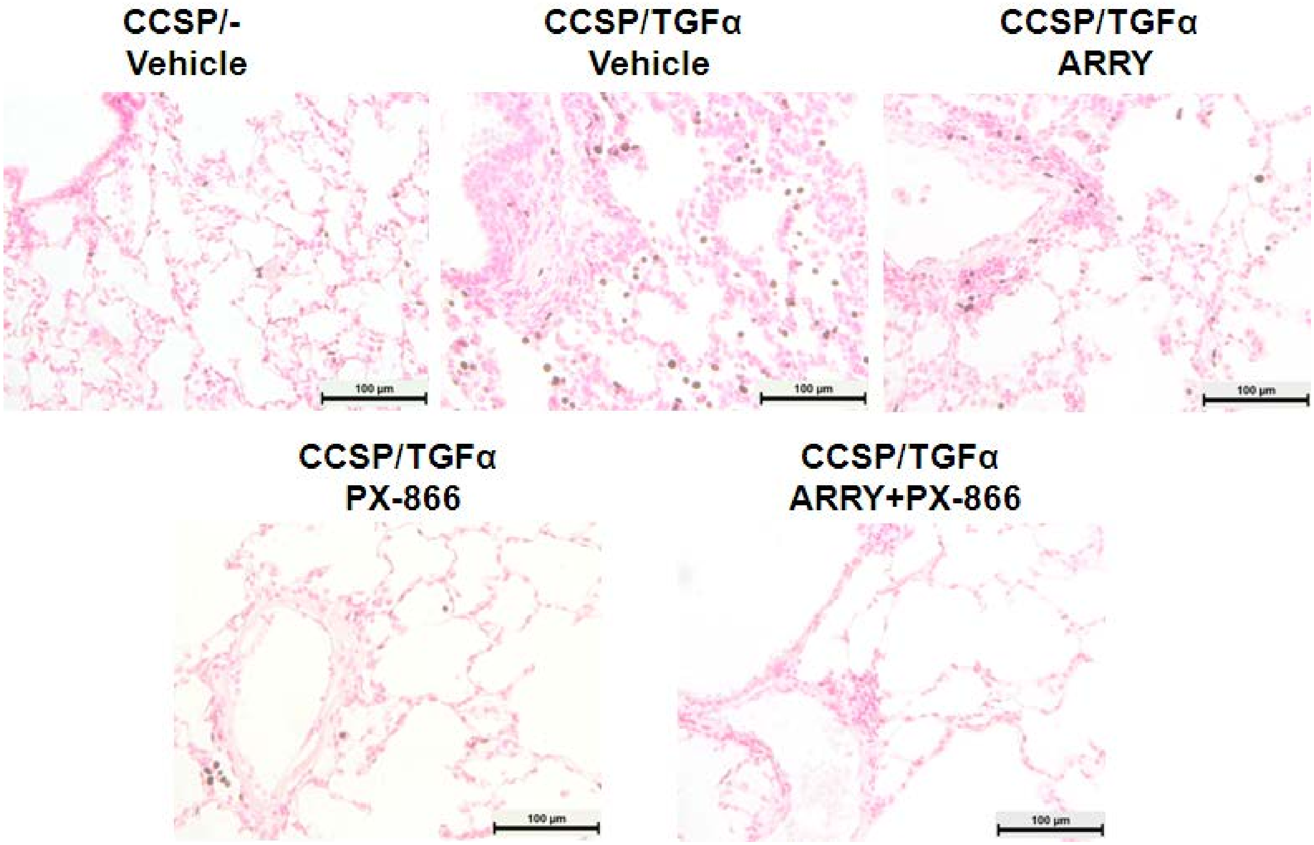

Figure S3: Expression of MYCN in the lungs of individuals with interstitial lung disease. Average Mycn mRNA levels are elevated in individuals with interstitial lung disease compared to controls and COPD. Mycn expression data was extracted from mRNA expression catalogue available in the database of Lung Genomic Research Consortium (LGRC) (https://www.lung-genomics.org/research)

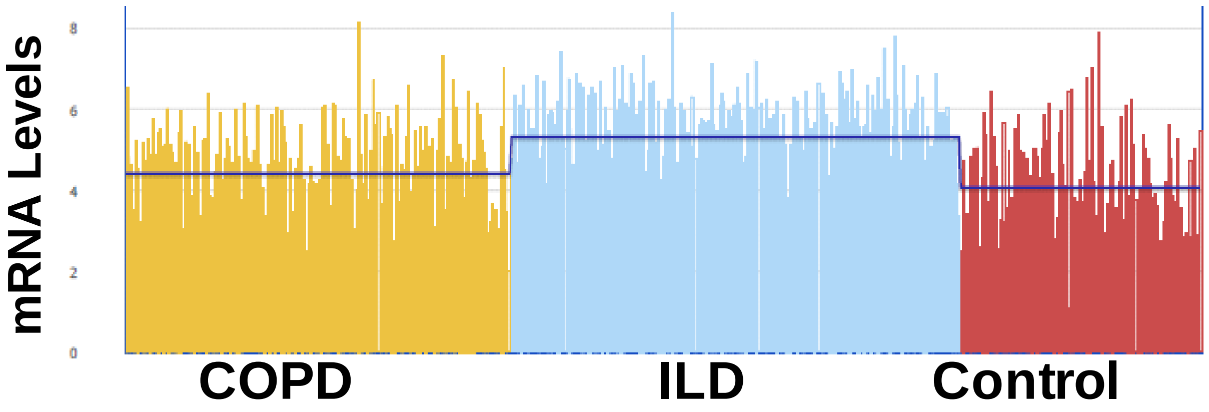

Supplement: Data S1 — (PDF) [file pone.0086536.s004.pdf]
